# Supplementary material for: Association between oral microbiome and seven types of cancers in East Asian population: a two-sample Mendelian randomization analysis
Source: Front Mol Biosci. 2023 Nov 21;10:1327893. doi: 10.3389/fmolb.2023.1327893 (PMC10702768; doi:10.3389/fmolb.2023.1327893)
Supplement: Supplementary file 1 [file DataSheet1.ZIP › Supplementary Materials/MR plots of saliva/saliva═╝/colorectal cancer/pheno.1428_to_colorectal cancer_forest.pdf]

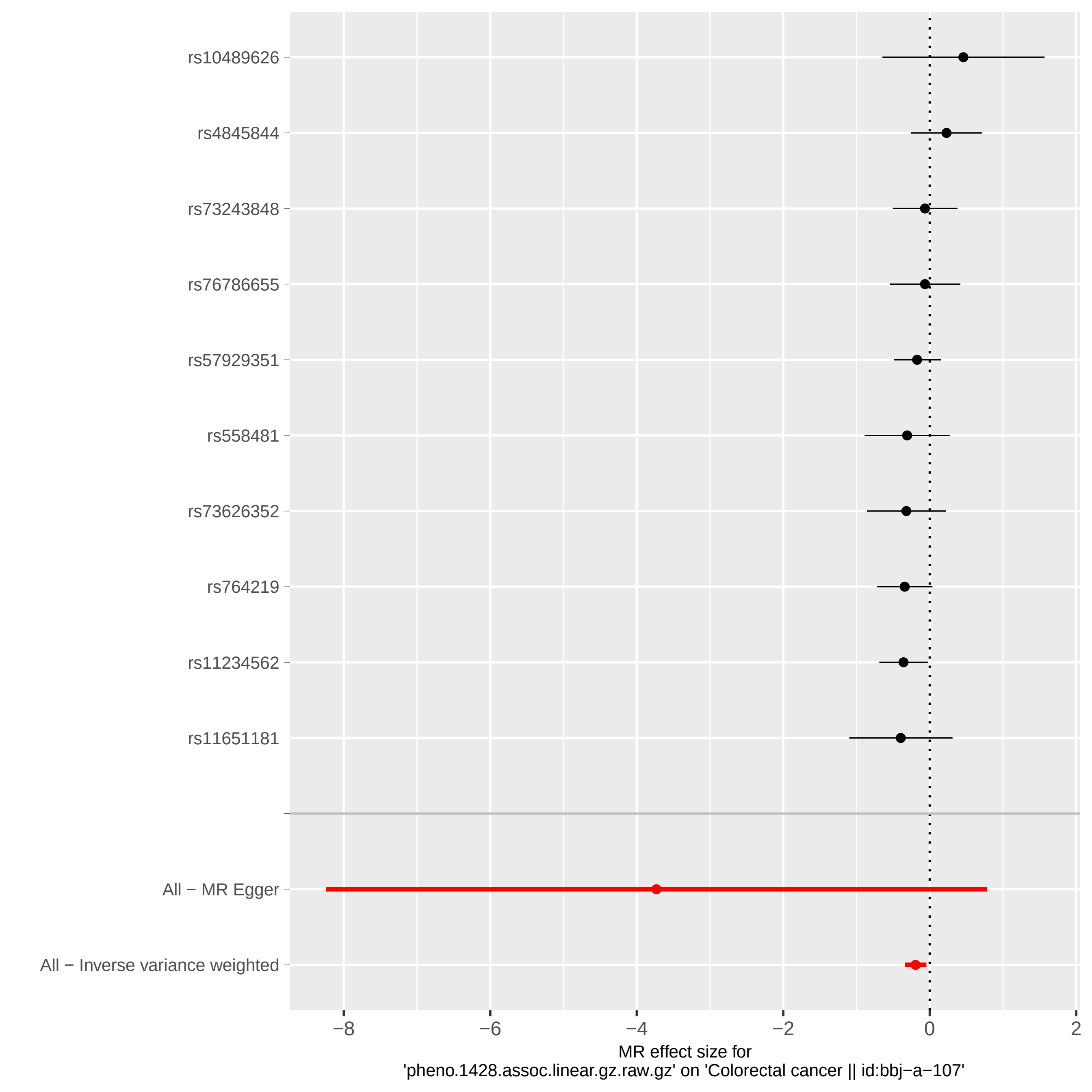
rs10489626
rs4845844
rs73243848
rs76786655
rs57929351
rs558481
rs73626352
rs764219
rs11234562
rs11651181
All − MR Egger
All − Inverse variance weighted
−8
−6
−4
−2
0
2
MR effect size for
'pheno.1428.assoc.linear.gz.raw.gz' on 'Colorectal cancer || id:bbj−a−107'
